# Injection Brou

**Published:** 1881-12

**Authors:** 


### INJECTION BROU.

The following is given by the *Med. Record* as the formula for this famed gonorrhœa injection : Sulphate of zinc, eight grains ; acetate of lead, fifteen grains ; tincture of catechu, two drachms ; aqueous tincture of opium, three ounces. The formula of the aqueous tincture of opium is known to but few pharmacists, and it is, therefore, not easily obtained. It is, however, not impossible that a dilution of tincture of opium would answer all purposes.

